# Automated MRI Lung Segmentation and 3D Morphologic Features for Quantification of Neonatal Lung Disease

**DOI:** 10.1148/ryai.220239

**Published:** 2023-10-25

**Authors:** Benedikt Mairhörmann, Alejandra Castelblanco, Friederike Häfner, Vanessa Koliogiannis, Lena Haist, Dominik Winter, Andreas Flemmer, Harald Ehrhardt, Sophia Stöcklein, Olaf Dietrich, Kai Förster, Anne Hilgendorff, Benjamin Schubert

**Affiliations:** From the Computational Health Center (B.M., A.C., D.W., B.S.), Institute for Lung Health and Immunity and Comprehensive Pneumology Center (F.H., L.H., A.H.), and Institute of AI for Health (D.W.), Helmholtz Zentrum München, Member of the German Center for Lung Research (DZL), Ingolstädter Landstrasse 1, 85764 Neuherberg, Germany; Department of Neonatology, Perinatal Center (F.H., A.F., K.F.), Department of Radiology (V.K., S.S., O.D.), and Center for Comprehensive Developmental Care (CDeCLMU) at the Interdisciplinary Social Pediatric Center, Dr. von Hauner Children’s Hospital (A.H.), Hospital of the Ludwig-Maximilian University, Munich, Germany; Department of General Pediatrics & Neonatology, Justus-Liebig-University, Member of the German Center for Lung Research (DZL), Giessen, Germany (H.E.); Division of Neonatology and Pediatric Intensive Care Medicine, Department of Pediatrics and Adolescent Medicine, University Medical Center Ulm, Ulm, Germany (H.E.); and Department of Mathematics, Technical University of Munich, Munich, Germany (B.S.).

**Keywords:** Bronchopulmonary Dysplasia, Chronic Lung Disease, Preterm Infant, Lung Segmentation, Lung MRI, BPD Severity Assessment, Deep Learning, Lung Imaging Biomarkers, Lung Topology

## Abstract

**Purpose:**

To analyze the performance of deep learning (DL) models for segmentation of the neonatal lung in MRI and investigate the use of automated MRI-based features for assessment of neonatal lung disease.

**Materials and Methods:**

Quiet-breathing MRI was prospectively performed in two independent cohorts of preterm infants (median gestational age, 26.57 weeks; IQR, 25.3–28.6 weeks; 55 female and 48 male infants) with (*n* = 86) and without (*n* = 21) chronic lung disease (bronchopulmonary dysplasia [BPD]). Convolutional neural networks were developed for lung segmentation, and a three-dimensional reconstruction was used to calculate MRI features for lung volume, shape, pixel intensity, and surface. These features were explored as indicators of BPD and disease-associated lung structural remodeling through correlation with lung injury scores and multinomial models for BPD severity stratification.

**Results:**

The lung segmentation model reached a volumetric Dice coefficient of 0.908 in cross-validation and 0.880 on the independent test dataset, matching expert-level performance across disease grades. MRI lung features demonstrated significant correlations with lung injury scores and added structural information for the separation of neonates with BPD (BPD vs no BPD: average area under the receiver operating characteristic curve [AUC], 0.92 ± 0.02 [SD]; no or mild BPD vs moderate or severe BPD: average AUC, 0.84 ± 0.03).

**Conclusion:**

This study demonstrated high performance of DL models for MRI neonatal lung segmentation and showed the potential of automated MRI features for diagnostic assessment of neonatal lung disease while avoiding radiation exposure.

**Keywords:** Bronchopulmonary Dysplasia, Chronic Lung Disease, Preterm Infant, Lung Segmentation, Lung MRI, BPD Severity Assessment, Deep Learning, Lung Imaging Biomarkers, Lung Topology

*Supplemental material is available for this article.*

Published under a CC BY 4.0 license.

See also the commentary by Parraga and Sharma in this issue.

## Graphical abstract

**Figure:**
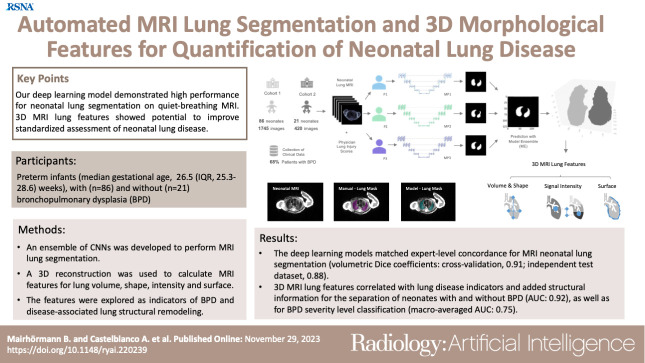

**Summary**

The deep learning model ensemble demonstrated high performance for segmentation of the neonatal lung in quiet-breathing MRI; automated three-dimensional MRI lung features showed potential for a standardized assessment of neonatal lung disease.

**Key Points**

■ The deep learning models matched expert-level concordance for MRI neonatal lung segmentation (volumetric Dice coefficients: cross-validation scheme, 0.91; independent test dataset, 0.88).
■ Three-dimensional (3D) MRI features correlated with lung disease indicators, including lung volume by birth weight and duration of mechanical ventilation (Spearman *r* = 0.735, *P* ≤ .001, *n* = 103) and MRI anteroposterior centroid displacement and anteroposterior ventilation inhomogeneity score (Spearman *r* = 0.516 for left lung, *P* ≤ .001).
■ The 3D MRI features added structural information for the classification of neonatal lung disease, separating neonates with and without bronchopulmonary dysplasia (BPD) (area under the receiver operating characteristic curve [AUC], 0.92 ± 0.02), mild versus severe BPD (AUC, 0.84 ± 0.03), and single-level BPD (AUC, 0.75 ± 0.01).

## Introduction

Preterm and term neonates may postnatally develop lung injury that can potentially evolve into chronic lung disease, also referred to as bronchopulmonary dysplasia (BPD) (1–4). The current diagnostic process relies solely on clinical observation, occasional chest radiography, and late-stage pulmonary function. This process has limited capacity to capture in-depth disease characteristics, resulting in reduced prognostic value. Thus, the development of much needed personalized treatment and monitoring strategies in this high-risk cohort depends on the implementation of sensitive, radiation-free imaging strategies and their standardized assessment to critically inform the diagnostic process by adding structural information (1,5,6).

The low sensitivity and diagnostic value of conventional chest radiography and the limitations of CT due to radiation exposure (7,8) resulted in the exploration of alternative imaging techniques such as MRI to characterize lung disease (9–13). The use of MRI is supported by its established role in the diagnosis of central nervous system abnormalities (14). Initial studies targeted lung volume measurements in MRI (9–11), whereas only a few studies explored the assessments of structural changes in the neonatal lung (12,15).

In the neonatal lung, MRI is technically challenged by small patient sizes, lower spatial resolution, and sensitivity to infant motion, resulting in blurring, ghosting, and other image artifacts (16); this demands expert knowledge to obtain qualitative and quantitative measurements (10,12). The reduced standardization due to interrater inconsistencies limits high-throughput MRI-based analysis and thus monitoring of neonatal lung disease.

We, therefore, developed a deep learning (DL)–based model to enable robust and standardized analysis of lung MRI in preterm neonates with and without BPD performed during quiet-breathing at near-term age. We combined recent advances in computational methods (2,17,18), that is, convolutional neural networks, to improve the applicability and robustness of DL methods for performing MRI lung segmentation in preterm infants. The obtained lung segmentations were used to compute three-dimensional (3D) MRI lung features that quantify lung volume and shape, pixel intensity distributions, and surface. We assessed these structural features for their added value in classifying infants according to the clinical diagnosis of BPD.

## Materials and Methods

### Study Cohort

**Figure 1:**
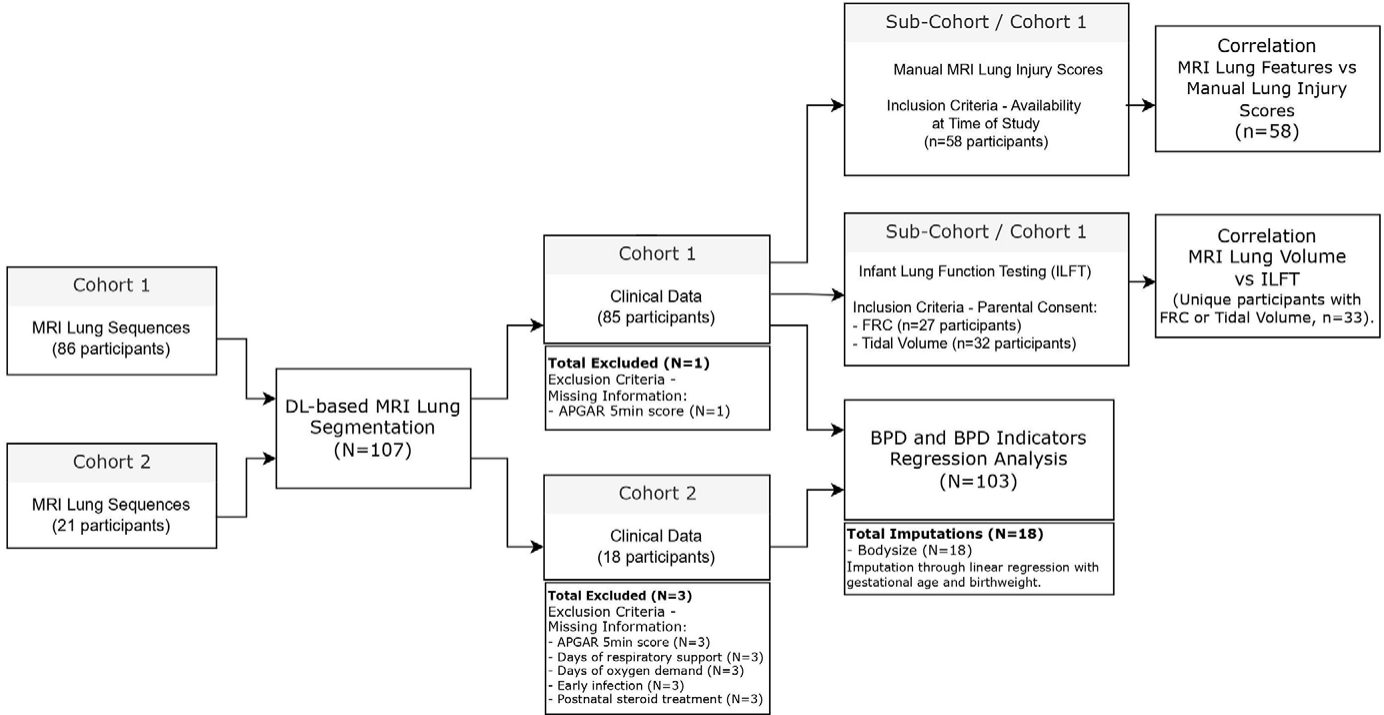
Data flow and participant exclusion process for the analyses performed in this study. *n* = number of participants analyzed. APGAR = appearance, pulse, grimace, activity, and respiration, BPD = bronchopulmonary dysplasia, DL = deep learning, FRC = functional residual capacity, ILFT = infant lung function testing.

We prospectively enrolled 107 preterm infants less than 32 weeks gestational age (GA) at birth (median age, 26.57 weeks; IQR, 25.3–28.6 weeks) with and without later development of BPD at two study sites (cohort 1: Perinatal Centre Hospital of the Ludwig-Maximilian University in Munich, Germany [*n* = 86]; cohort 2: Universitätsklinikum Giessen und Marburg, Germany [*n* = 21]). We performed 3-T lung MRI near term age during quiet breathing after obtaining informed parental consent (ethics vote cohort 1 [EC#195–07] and cohort 2 [EC#135–12]). MR images were acquired during quiet sleep at room air breathing in unsedated infants (cohort 1) or under light sedation with chloral hydrate (30–40 mg/kg orally, cohort 2) (Appendix S1, section 1). BPD was diagnosed in 73 infants and was classified into three severity grades based on the National Institutes of Health consensus definition summarized by Jobe and Bancalari (1) as mild (*n* = 42; requirement of supplemental oxygen for 28 days, no need for oxygen supplementation at 36 weeks postmenstrual age), moderate (*n* = 11; requirement of supplemental oxygen for 28 days and oxygen supplementation <30% fraction of inspired oxygen [FiO_2_] at 36 weeks postmenstrual age), and severe (*n* = 20; requirement of supplemental oxygen for 28 days and oxygen supplementation >30% FiO_2_ at 36 weeks postmenstrual age and/or positive pressure ventilation or continuous positive pressure). Thirty-four infants did not develop BPD. Clinical data were obtained from participants of both cohorts (*n* = 103) (Table 1). Four infants were excluded from the regression analysis due to missing clinical parameters (Fig 1).

**Table 1:**
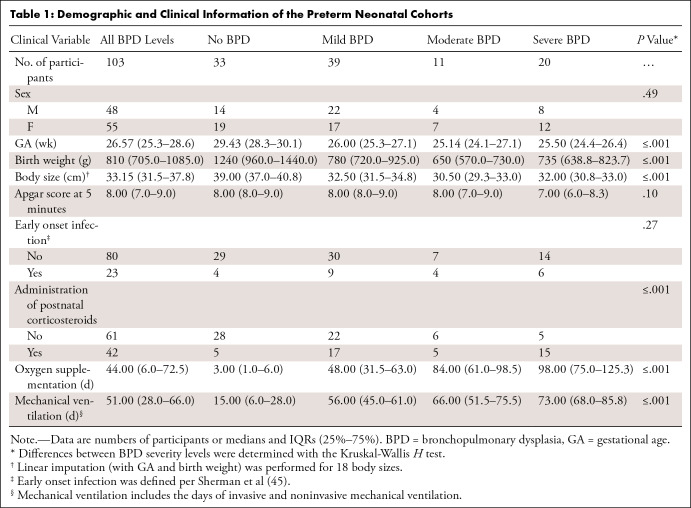
Demographic and Clinical Information of the Preterm Neonatal Cohorts

### MRI Protocols and Annotations

Axial MR images were obtained near term age (median, 35.95 weeks; IQR, 33.7–37.9 weeks) using 3-T MRI scanners (Siemens Skyra [cohort 1] and Siemens Verio [cohort 2]), with a size-adapted number of coil elements from the 32-channel spine array coil, an 18-channel flexible body array coil, and a 20-channel head-and-neck array coil (Appendix S1, section 2). A T2-weighted half-Fourier-acquired single-shot fast spin-echo sequence with an echo time of 57 msec was used, providing T2-weighted signal and contrast for lung structural assessment (19). Spatial resolution was 1.3 × 1.9 mm^2^ in plane, with a section thickness of 4 mm and a section gap of 0.4 mm.

Manual lung annotation was performed independently by three trained physicians, including a senior radiologist (V.K., with ≥5 years of experience) and medical students (F.H., L.H.), referred to as "raters" in the analysis (Appendix S1, section 2), using ITK-SNAP (20). Pseudonymization of images and clinical information was performed for all participants. MR images were automatically cropped to 128 × 128 pixels for model training.

### Lung Injury Score

Standardized scoring of MR images for lung structural changes was performed independently by a senior neonatologist and a senior radiologist, both blinded to the clinical diagnosis (Appendix S1, section 2), in a subcohort of infants from cohort 1 (*n* = 58; median GA at birth, 26.9 weeks [IQR, 25.4–28.7 weeks]; median birth weight, 817.5 g [IQR, 712.5–1077.5 g]; 22 without BPD, 16 with mild BPD, seven with moderate BPD, and 13 with severe BPD), as previously described (21). Variables, scored on a five-point Likert scale, included interstitial enhancement, caudocranial and anteroposterior gradients, emphysema and atelectasis, and airway accentuation (Appendix S1, section 2).

### Infant Lung Function Testing

Infant lung function testing was performed in a subcohort of preterm infants from cohort 1 (*n* = 33; median GA at birth, 28.0 weeks [IQR, 25.4–30.0 weeks]; median birth weight, 920 g [IQR, 750–1300 g]; 17 with no BPD, 10 with mild BPD, two with moderate BPD, and four with severe BPD) at near-term age (median age, 36.6 weeks [IQR, 34.6–38.7 weeks]) and included tidal breathing analysis and functional residual capacity at body plethysmography (22).

### DL MRI Lung Segmentation Model

**Figure 2:**
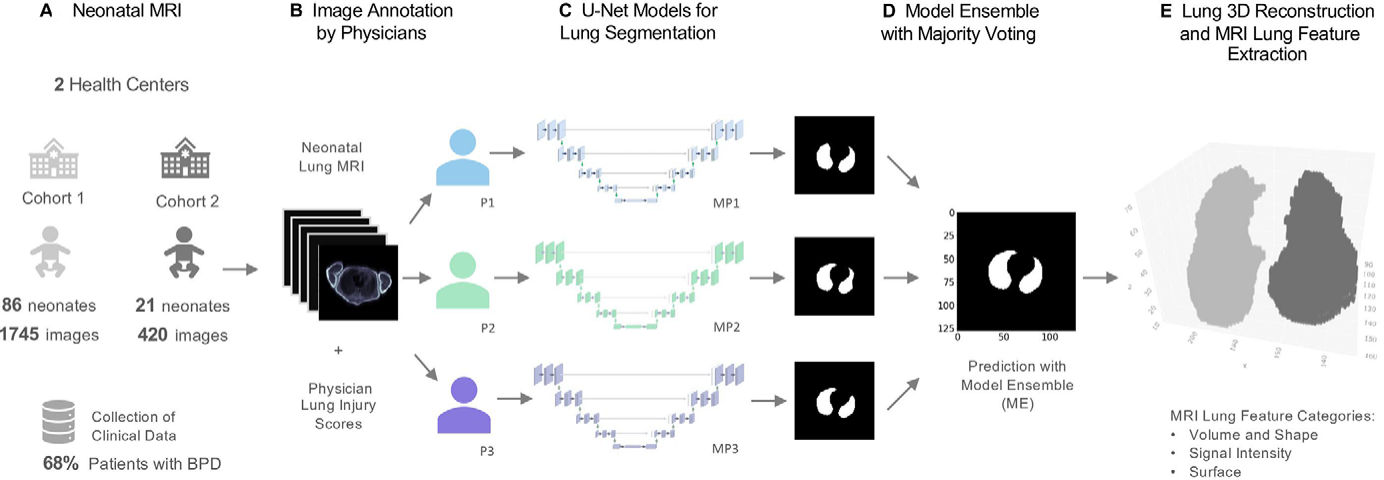
MRI-based neonatal lung segmentation and automated MRI analysis. **(A)** Clinical study including preterm infants with and without bronchopulmonary dysplasia (BPD). Free-breathing neonatal MRI was performed at mean gestational age of 37 weeks ± 5.8. **(B)** Manual MRI annotation of the lung was performed by three trained physicians (physician 1 [P1], physician 2 [P2], and physician 3 [P3]). MRI morphologic injuries (eg, emphysema, fibrosis, ventilation inhomogeneity) were scored by two trained physicians. **(C, D)** U-Net deep learning models (MP1, MP2, MP3) were trained for lung segmentation, and a final lung-mask prediction was calculated with an ensemble of the models (ME) through majority voting. **(E)** Lung volume three-dimensional (3D) reconstruction and automated calculation of 78 lung morphologic 3D descriptors.

We trained a set of U-Net convolutional neural network models (23) to perform two-dimensional lung segmentation on the collected neonatal MR images, with each model (models 1–3) based on the manual annotations of every rater, and combined them through pixelwise majority voting to a model ensemble (ME) (Fig 2).

U-Net architecture and hyperparameters are available in Appendix S1, section 3, in Tables S2 and S3, and in the code repository (*https://github.com/SchubertLab/NeoLUNet*). The Instant-DL framework was adapted for model training (24). In addition, we trained multiple 3D U-Net models (25) to investigate their performance in comparison with the two-dimensional models (Appendix S1, section 3).

To generate unbiased training in cohort 1, a set of k models were trained in a leave-one-patient-out cross-validation scheme. Additionally, a model trained with all the data from cohort 1 was validated in cohort 2. Lung segmentation performance was measured with the volumetric Dice coefficient (VDC) (26). The ME prediction was evaluated by comparing its VDC performance against the manual segmentation of each physician (physician 1, physician 2, physician 3), as well as against a physician consensus obtained by pixelwise majority voting. The average interrater VDC concordance between physicians and performance of each individual model were evaluated for reference.

After segmentation, a 3D representation of the lung was created by thresholding the predicted lung masks and finding the connected voxels in 3D. Lungs were rotated to a common reference frame (Appendix S1, section 4). Lung volumes obtained from the U-Net segmentations were validated through comparison with volumes obtained from manual segmentations.

### Morphologic MRI Lung Features

We automatically extracted a set of 78 MRI lung features from 3D reconstructions of the lung, thereby representing the morphologic features of the left and right lung, based on the analysis described by Waibel et al (27). In addition, we proposed a set of pixel-intensity features using scikit-Image 0.19.2 (28) to investigate their potential for describing lung injury in the challenging case of neonatal lung MRI. MRI lung features were grouped into the following categories: volumetric features (*n* = 38) describing volumes, axis lengths, centroids, inertias, and moments for each 3D axis; intensity features (*n* = 30), including intensity-weighted centroids and descriptive statistics of the distribution of pixel intensities in the lung; and surface features (*n* = 10) quantifying surface area, roughness, and convexity (Table S4). An exploratory analysis was performed to investigate the correlation between MRI lung features and BPD indicators, clinical parameters, and lung injury scores (Appendix S1, section 5). For reference, we additionally extracted standard radiomic features (120 per lung) that are used for medical imaging in adult patients using the Python package PyRadiomics 3.0.1 (Appendix S1, section 9) (29).

### BPD Severity Classification Models

The integrated potential of MRI lung features to complement BPD disease classification by adding information on lung volume and structure was estimated by training multiple regression models for BPD severity (ie, mild, moderate, severe) or the expression of BPD indicators (ie, duration of mechanical ventilation or oxygen exposure) using three groups of explanatory variables: *(a)* 78 MRI lung features (hereafter, L) and clinical BPD risk factors divided into *(b)* patient attributes (hereafter, P) (ie, GA, birth weight, body size, sex) and *(c)* postnatal clinical adaptation (hereafter, C) (ie, 5-minute Apgar score, early-onset infection, steroid treatment).

Random forest (30) and logistic regression models with Elastic Net (31) regularization were trained to perform binomial classification of two scenarios (no BPD vs BPD; no or mild BPD vs moderate or severe BPD) and multinomial classification (no BPD, mild BPD, moderate BPD, severe BPD), using scikit-learn v.1.1.1 (32). The models were trained using different combinations of the grouped explanatory variables (L, PC, PCL) to thereby estimate the added value of the MRI lung features to characterize BPD.

To optimize the hyperparameters with a randomized search (Appendix S1, section 6; Table S5), we used a nested cross-validation scheme where the model performance was estimated with 10 repetitions. A stratified fivefold train-test split was used for inner and outer cross-validation loops.

We also evaluated model performance when applying univariate feature selection or principal component analysis to the input features. Ultimately, a set of logistic regression and random forest models were trained both with and without feature selection, with the aim of finding the best combination of model, hyperparameters, and groups of features (Appendix S1, section 6).

For the continuous BPD indicators, regression models (ie, Poisson and random forest) were trained to estimate the duration of required respiratory support and oxygen supplementation, using the same nested cross-validation and feature selection schemes.

### Statistical Analysis

To identify statistical differences between cohorts and BPD severity groups, normality tests (D’Agostino and Pearson) were followed by the Kruskal-Wallis *H* test with Bonferroni correction. MRI lung segmentation performance across cohorts, disease severity, and lung injury scores was evaluated with normality and Kruskal-Wallis testing. DL-based lung volumes were correlated with other lung volume estimators using normality tests and subsequent Pearson or Spearman correlations. For the exploratory MRI lung feature analysis and correlation heat map, Spearman correlations with Bonferroni corrections were calculated, and Kruskal-Wallis and pairwise Mann-Whitney *U* tests were used to determine the individual features’ potential for disease grade or lung injury score discrimination. Performance of BPD classification models was calculated via the area under the receiver operating characteristic curve (AUC). Model performances were compared using Kruskal-Wallis and pairwise Mann-Whitney *U* tests. Significant differences were reported when *P* < .05. Statistical analyses were performed with Python 3.7 and SciPy 1.10.1 (Python Software Foundation).

## Results

### Participant Characteristics

Demographic and clinical variables of the study cohorts (median GA, 26.57 weeks [IQR, 25.3–28.6 weeks]; 55 female and 48 male infants) (Table 1) were found comparable between cohorts (Table S1) and within the range of previous published studies (4,33).

### DL Enables Robust MRI Neonatal Lung Segmentation across Disease Grades

**Figure 3:**
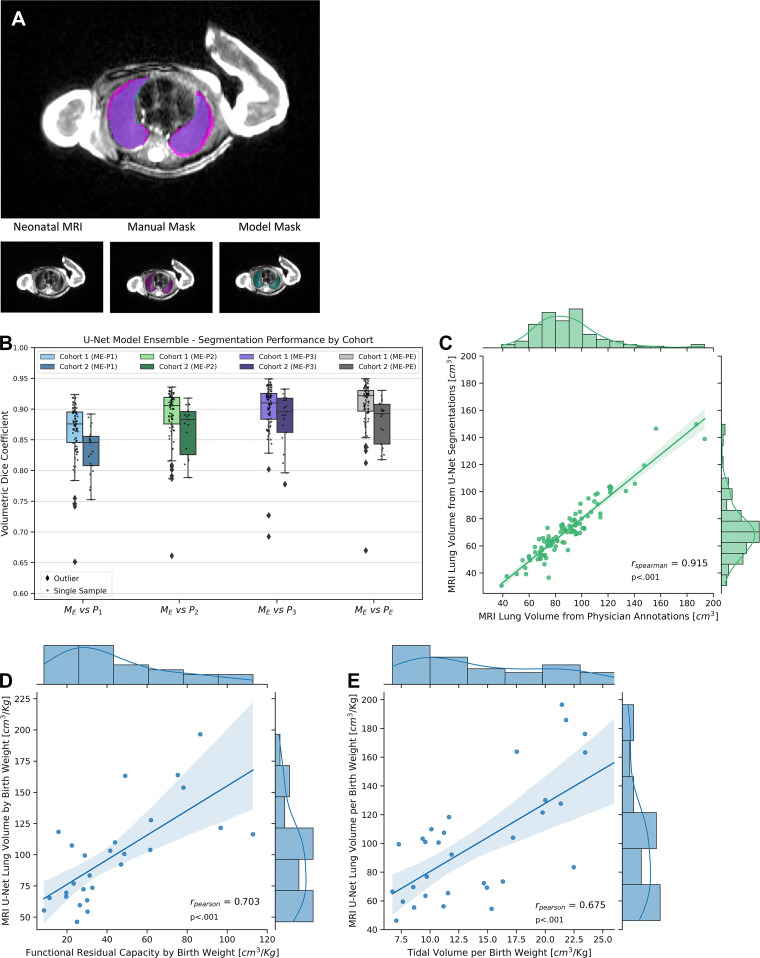
Lung segmentation and lung volume analysis. **(A)** MRI lung segmentation sample with manual annotation (magenta) and machine learning model–generated lung masks (cyan). **(B)** Plot shows lung segmentation performances for manual physician-based lung annotations (physician 1 [P1], physician 2 [P2], physician 3 [P3]), and the model ensemble (ME) with majority voting; results are separated for cohort 1 and cohort 2. Boxes represent IQR (25th–75th percentile), median value is the horizontal midline, whiskers extend to data points within ± 1.5 IQR from each quartile, outliers are plotted as diamonds. **(C)** Graph shows MRI lung volume from the U-Net model ensemble segmentations versus estimated lung volume from manual segmentations (*n* = 107). **(D)** Graph shows functional residual capacity per birth weight versus MRI model ensemble lung volume per birth weight (*n* = 27). **(E)** Graph shows tidal volume per birth weight versus MRI model ensemble lung volume per birth weight (*n* = 32). The shaded area in **C–E** corresponds to the regression 95% CI, and axis plots show univariate histograms and probability density curves.

The DL lung segmentation ME achieved high segmentation performance (Fig 3A), measured as the VDC against each rater (mean VDC = 0.859 ± 0.046 [SD] for ME vs physician 1, 0.886 ± 0.044 for ME vs physician 2, and 0.897 ± 0.043 for ME vs physician 3) (Fig 3B). This was in line with the interrater concordance (mean VDC = 0.877 ± 0.038 for physician 1 vs physician 2, 0.872 ± 0.036 for physician 1 vs physician 3, and 0.885 ± 0.038 for physician 2 vs physician 3). The ME compared with the physician consensus showed a mean VDC of 0.903 ± 0.040 (Fig 3B), confirming human-level accuracy of the artificial intelligence–based segmentation. The VDC between cohorts was statistically different (Mann-Whitney *U* = 422, *P* ≤ .001) by 0.028 points (cohort 1: cross-validation, 0.908 ± 0.039; cohort 2: test dataset, 0.880 ± 0.036). The performance of individual models was comparable to the corresponding raters (Fig S1A; Appendix S1, section 7). The 3D U-Net models reached a performance of 0.793 ± 0.097 VDC, distinctly below the two-dimensional U-Net models (Appendix S1, section 3).

We found an effect of image quality on segmentation performance for both manual and automated segmentations (Fig S1B) and lower image qualities in cohort 2 (Mann-Whitney *U* = 462, *P* ≤ .001). In contrast, we found no evidence of a difference in the model’s segmentation performance between BPD severity grades (Fig S1C) or lung injury scores (Fig S2).

Reflecting the robust performance of the segmentation models, manual and DL-based computed MRI lung volumes (Fig 3C) were highly correlated (Spearman *r *= 0.915, *P* ≤ .001, *n* = 107).

### MRI Lung Features Correlate with BPD Severity, Disease Indicators, and Lung Injury Scores

**Figure 4:**
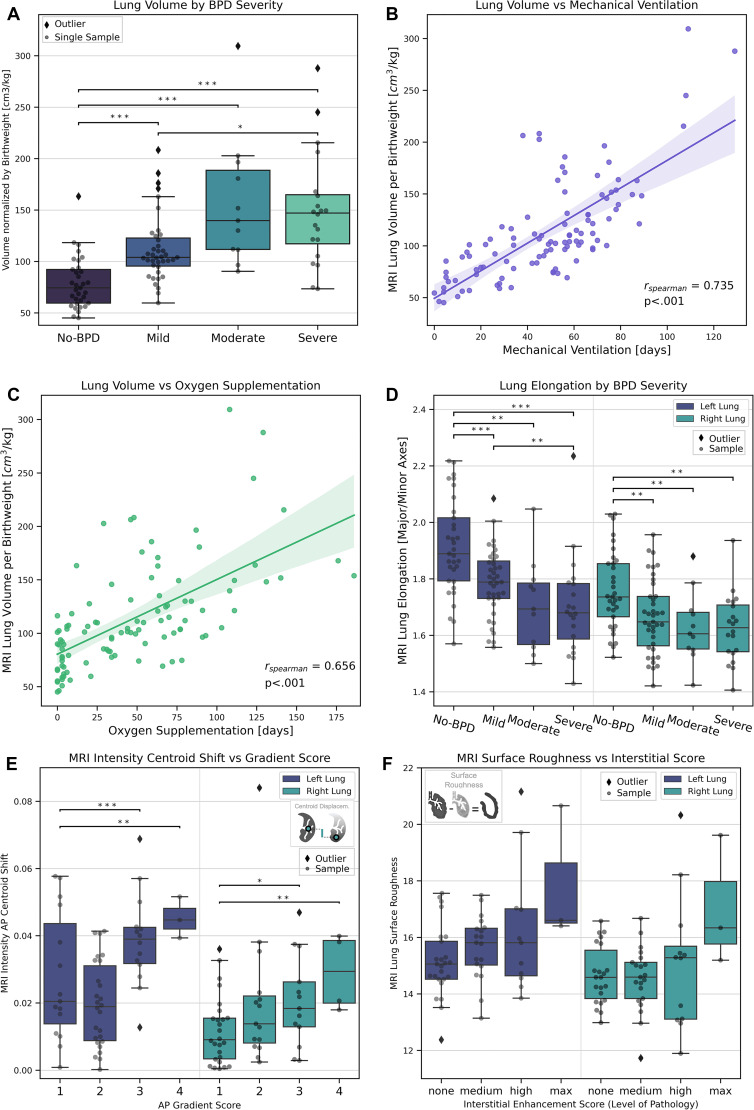
Correlation of MRI lung features with bronchopulmonary dysplasia (BPD) severity and lung injury scores. **(A)** Plot shows predicted lung volume normalized by birth weight against BPD severity grades (*n* = 103). **(B)** Graph shows correlation of lung volume based on model ensemble segmentations normalized by birth weight against duration of mechanical ventilation (in days) (*n* = 103). **(C)** Graph shows correlation of lung volume based on model ensemble segmentations normalized by birth weight with duration of oxygen supplementation (in days) (*n* = 103). **(D)** Plot shows lung elongation (major axis/minor axis) by BPD severity for right and left lungs (*n* = 103). The shaded area in **B** and **C** corresponds to the regression 95% CI. **(E)** Plot shows MRI lung intensity anteroposterior (AP) centroid displacement versus anteroposterior gradient score for ventilation inhomogeneity (*n* = 58). **(F)** Plot shows MRI lung volumetric surface roughness versus interstitial lung injury score for fibrosis (*n* = 58). max = maximum. Differences were tested with the Kruskal-Wallis *H* test with Bonferroni multiple test correction, and pairwise comparisons were performed with the Mann-Whitney *U* test (* = *P* ≤ .05, ** = *P* ≤ .01, *** = *P* ≤ .001). Boxes in **A** and **D–F** represent IQR (25th–75th percentile), median value is the horizontal midline, whiskers extend to data points within ± 1.5 IQR from each quartile, outliers are plotted as diamonds.

Our exploratory analysis demonstrated significant correlations of multiple MRI lung features with BPD severity, indicators of BPD, and lung injury scores. When comparing MRI lung volume with variables of infant lung function testing, direct estimators of lung function, we observed a positive correlation with functional residual capacity (Pearson *r* = 0.703, *P* ≤ .001, *n* = 27) (Fig 3D) and tidal volume (Pearson *r* = 0.675, *P* ≤ .001, *n* = 32) (Fig 3E), with all three normalized by birth weight. The normalized MRI lung volume allowed for discrimination between BPD severity grades (Kruskal-Wallis test, *H* = 42.17; *P* ≤ .001; *n* = 103), as indicated by pairwise comparisons (Fig 4A). Moreover, MRI lung volume normalized by birth weight correlated with the continuous BPD indicators duration of mechanical ventilation (Spearman *r* = 0.735, *P* ≤ .001, *n* = 103) (Fig 4B) and oxygen supplementation (Spearman *r* = 0.656, *P* ≤ .001, *n* = 103) (Fig 4C).

The volumetric feature “lung elongation” helped differentiate BPD severity levels (left lung: Spearman *r* = −0.502, *P* ≤ .001; right lung: *r* = −0.370, *P* ≤ .001; *n* = 103) (Fig 4D) and correlated with the duration of mechanical ventilation (left lung: Spearman *r* = −0.577, *P* ≤ .001; right lung: *r* = −0.426, *P* ≤ .001; *n* = 103).

**Figure 5:**
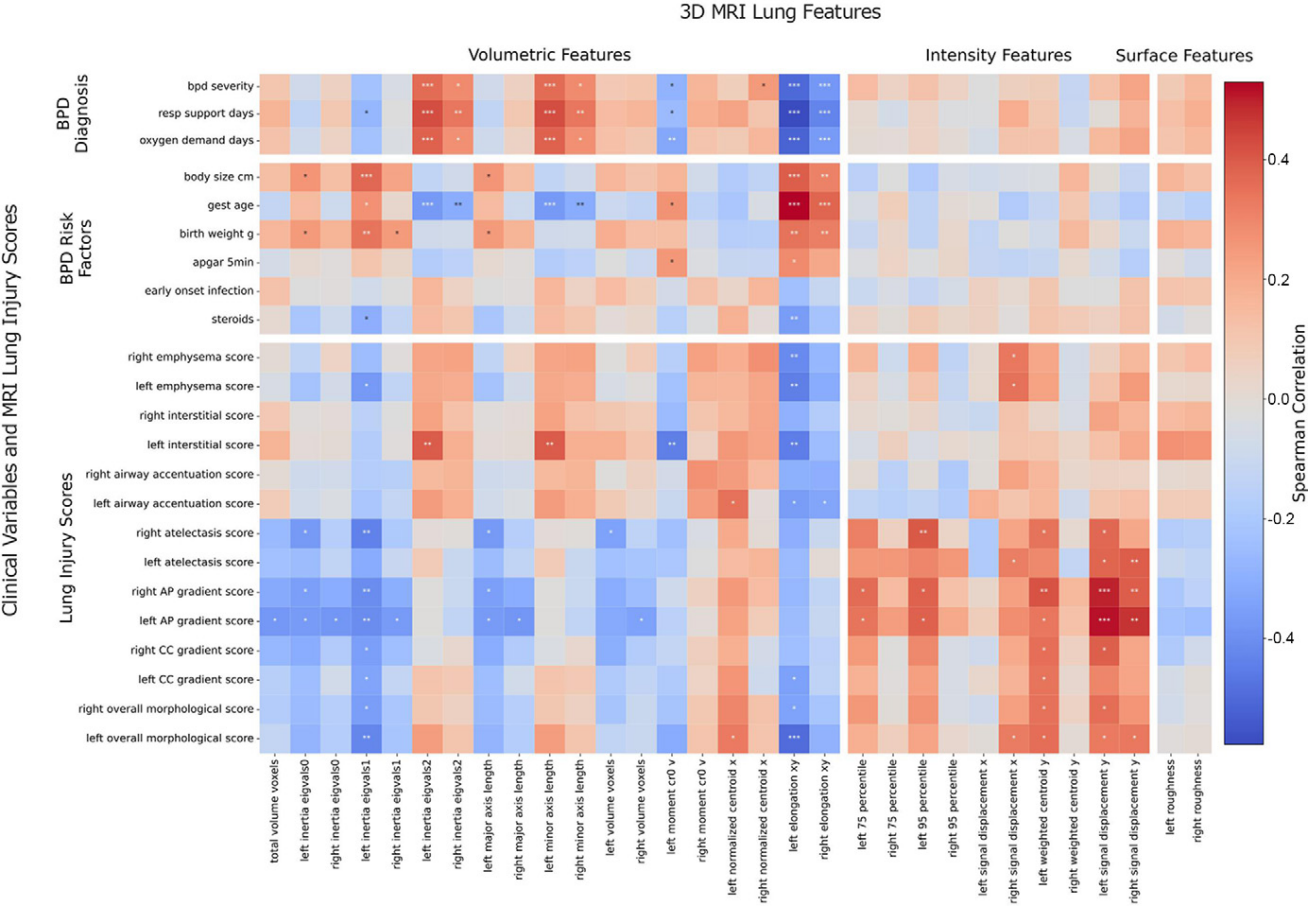
Correlation matrix of three-dimensional (3D) MRI lung features with clinical variables (bronchopulmonary dysplasia [BPD] diagnosis variables and BPD risk factors) and lung injury scores. MRI lung features are grouped by feature type (volumetric, intensity, and surface). A subset of morphologic features with the highest Spearman correlations is shown. Statistical significance is annotated based on Spearman correlations with multiple test Bonferroni correction (* = *P* ≤ .05, ** = *P* ≤ .01, *** = *P* ≤ .001). AP = anteroposterior, CC = craniocaudal, gest = gestational, resp = respiratory.

We also found positive correlations of MRI lung features with variables of the lung injury score, such as between MRI anteroposterior centroid displacement and the anteroposterior gradient (left lung: Spearman *r* = 0.516, *P* ≤ .001; right lung: *r* = 0.395, *P* = .0099, *n* = 58) (Fig 4E) as well as between lung surface roughness and interstitial enhancement (left lung: Spearman *r* = 0.273, *P* = .11, *n* = 58) (Fig 4F); all feature correlations are shown in Figure S3 and Appendix S1, section 8; the highest correlations are shown in Figure 5.

### MRI Lung Features Complement Stratification Value of Clinical BPD Estimators

**Figure 6:**
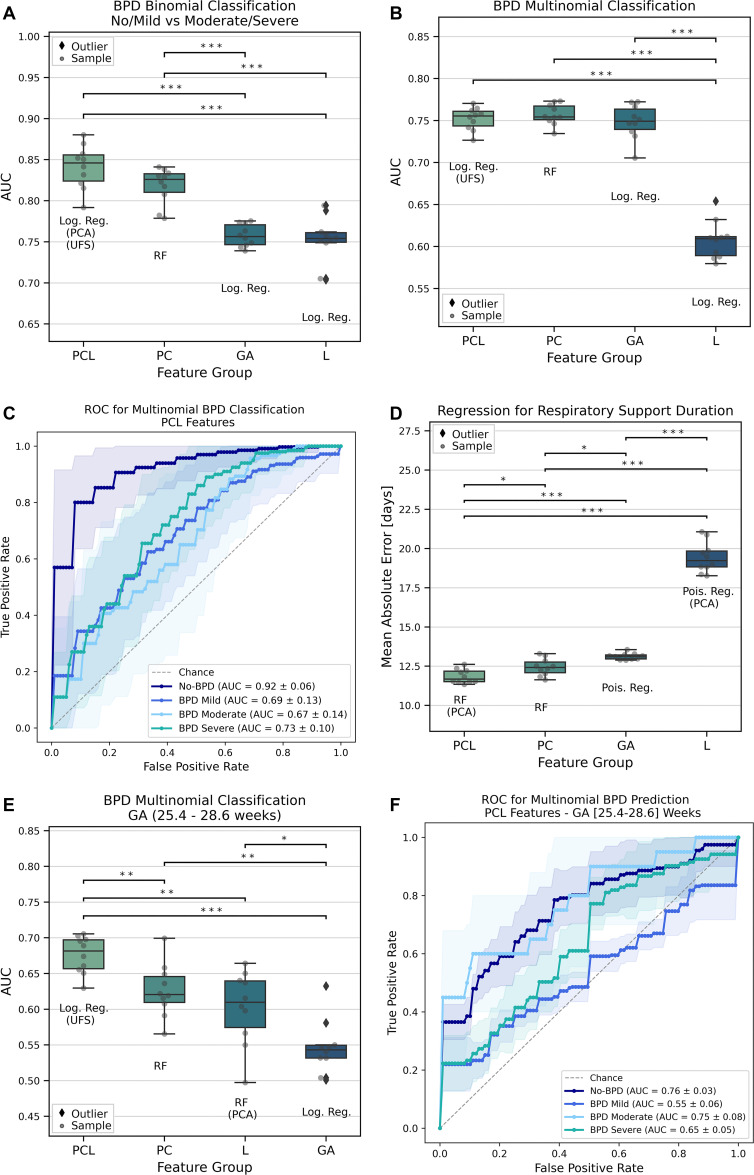
Bronchopulmonary dysplasia (BPD) classification with best performing models by feature group (GA = gestational age, L = 78 MRI automated lung features, PC = patient and clinical variables, PCL = patient, clinical, and lung features). **(A)** Plot shows BPD binomial classification performance (no or mild vs moderate or severe). **(B)** Plot shows BPD multinomial classification performance (no, mild, moderate, severe). **(C)** Graph shows BPD multinomial receiver operating characteristic (ROC) curve for the best model with PCL features. **(D)** Plot shows regression performance for duration of respiratory support. **(E)** Plot shows BPD multinomial classification performance for patients with GA between 25.4 and 28.6 weeks. **(F)** Graph shows BPD multinomial receiver operating characteristic curve (ROC) for the best model with PCL features with GA (25.4–28.6 weeks). AUC = area under the receiver operating characteristic curve, Log. Reg. = logistic regression, PCA = principal component analysis, RF = random forest, UFS = univariate feature selection. Differences were tested with the Kruskal-Wallis *H* test with Bonferroni multiple test correction, and pairwise comparisons were performed with the Mann-Whitney *U* test (* = *P* ≤ .05, ** = *P* ≤ .01, *** = *P* ≤ .001). Boxes in **A, B, D,** and **E** represent IQR (25th–75th percentile), median value is the horizontal midline, whiskers extend to data points within ± 1.5 IQR from each quartile, and outliers are plotted as diamonds.

In binary BPD severity classification, all models showed comparable performance for separating infants with BPD from those without BPD (AUC = 0.92). However, for the separation of no and mild BPD from moderate and severe BPD, the inclusion of MRI lung features in the PCL model improved the average AUC by 0.08 when compared with GA alone and by 0.02 when compared with PC (Fig 6A, Table 2).

**Table 2:**
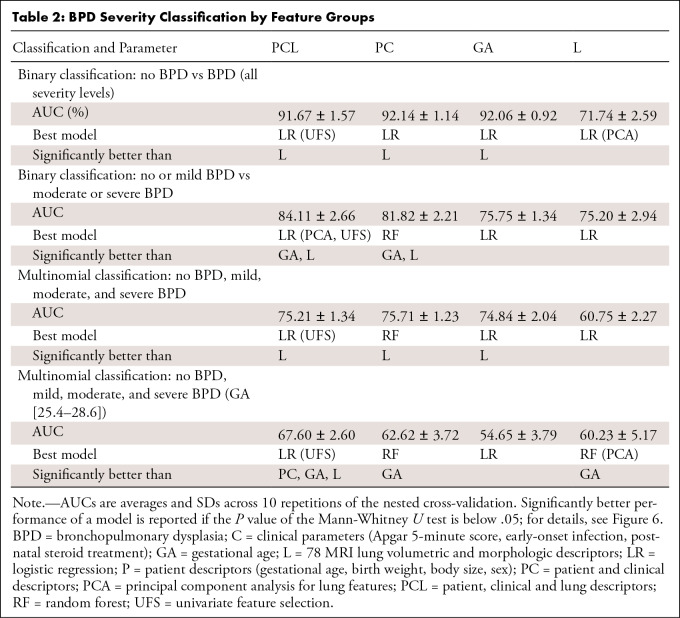
BPD Severity Classification by Feature Groups

The multiclass classification of BPD severity showed comparable performance for the PCL and PC models, with macro-weighted AUCs of 0.75 and 0.76, respectively (Table 2, Fig 6B). The individual class performance for the best PCL model was highest in the no-BPD class (AUC = 0.92), with similar AUC performances in the remaining classes (AUC = 0.67–0.73) (Fig 6C).

To specifically analyze the subgroup of extremely premature infants where clinical features show less discriminative power, we re-evaluated the model performance in a subgroup that included the most immature participant without BPD and the most mature infants with severe BPD (GA between 25.4 and 28.6 weeks; *n* = 50). Here, GA did not sufficiently discriminate for disease severity (AUC = 0.55), whereas the inclusion of lung MRI features significantly improved the separation of BPD grades, with an increase in performance of the PCL model by 0.05 versus PC and 0.13 versus GA (Table 2; Fig 6E, 6F).

Permutation feature importance analysis (100 repetitions) on the best logistic regression model trained with all the features (PCL) and data points (*n* = 103) revealed higher importance scores of 16 MRI lung features, next to GA and birth weight (Fig S4A). In the random forest model, 17 MRI lung features showed relevance for BPD classification (Fig S4B). Features identified in both analyses included lung volumes, lung elongation, and signal intensity centroids.

We investigated the models’ performance when classifying by BPD indicators, that is, duration of mechanical ventilation and oxygen supplementation, as continuous variables (Table 3). For the duration of respiratory support, the PCL model achieved the lowest mean absolute error over all feature groups (average mean absolute error, 11.85 days for PCL model, 12.45 days for PC model, and 13.12 days for GA model) (Fig 6D). For the duration of oxygen supplementation, the PCL model showed a similar performance to the PC and GA models, with an average mean absolute error of 23.88 days.

**Table 3:**
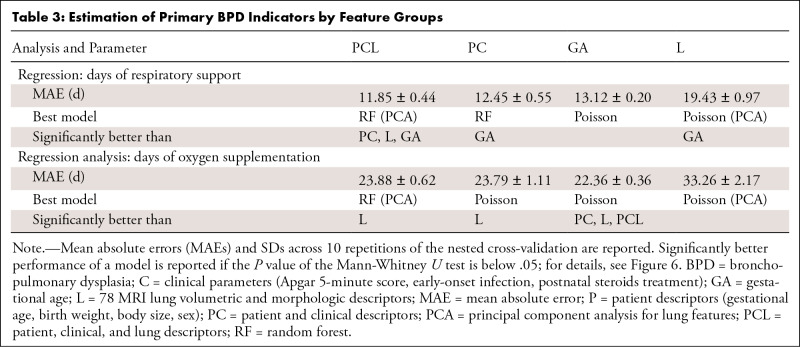
Estimation of Primary BPD Indicators by Feature Groups

PCL models using our MRI lung features showed comparable BPD classification performance to models using standard radiomic features. We found an AUC increase of 0.01 when combining both the radiomic and our MRI lung features compared with using only radiomic features and an increase of 0.03 in the same comparison within the subgroup with GAs between 25.4 and 28.6 weeks (Fig S5; Tables S6, S7; Appendix S1, section 9).

## Discussion

To our knowledge, we are the first to successfully apply DL for accurate lung segmentation on lung MR images in healthy neonates and those with lung disease (VDC, 0.91). We found significant correlations of volumetric and structural MRI lung features with clinically relevant disease indicators and demonstrated their potential for stratification of disease severity (AUC, 0.92 for binary classification and 0.75 for multiclass classification).

With low variability across disease grades and high accuracy, the performance of the segmentation models outweighed individual manual annotations, indicating their ability to contribute to the standardization of lung MRI analysis in neonates, a process that is challenged by small organ size, motion artifacts, and blurring. The use of a unique neonatal cohort with multiexpert annotations and extensive hyperparameter tuning resulted in a DL model ensemble that overcame limitations of scalability and sensitivity reported by previous studies on MRI lung segmentation. Heimann et al (34) used lung shape appearance models to perform free-breathing MRI lung segmentation in a cohort of 32 children, and the reported VDC was only 0.85, possibly due to reduced cohort size. Other studies achieved a segmentation overlap of 0.94–0.95 using lung region-growing–based methods and convolutional neural networks in MR images from adult patients while applying breath-holding maneuvers (35,36). Other adult MRI lung segmentation methods reported VDCs in the range of 0.82–0.86 (37,38). In contrast, our ensemble model achieved equal or superior performance when compared with models designed for controlled acquisition protocols in adult lungs, with an average performance VDC of 0.90. We demonstrate robust performance under lung disease conditions, corroborating the potential for clinical application. Differences in segmentation performance between cohorts can be explained by the significantly lower image qualities of cohort 2. The accurate estimation of neonatal lung volumes by our pipeline (compared with manual annotations, *r* = 0.92) were close to the correlation levels of MRI lung volume extractions in adults (*r *= 0.98) (35).

We found that MRI lung volume normalized by birth weight was representative of direct estimators of lung function, that is, tidal volume and functional residual capacity, validating the DL-based volumes with variables independent of imaging. In addition, we showed that MRI lung volumes normalized by birth weight sensitively represent the variability in BPD severity grades and BPD indicators, with the elevation in lung volume in disease being in line with previous studies (10,12). In agreement with these findings, lung elongation allowed for the identification of BPD cases, likely reflecting ventilation inhomogeneity and consequences of long-term ventilatory support. High correlations of structural markers with lung injury scores, such as intensity-weighted centroid displacement with anteroposterior gradients*,* reflect their potential to detect ventilation inhomogeneities. Likewise, the positive association between lung surface irregularities and the lung injury score for interstitial enhancement indicates the features’ potential to identify structural remodeling in the BPD lung, in line with results obtained in infant (13) and adult (39) lung fibrosis. Our approach acknowledges the potential of MRI lung features as interpretable, quantitative markers of lung structural injuries in neonates, with the prospect of representing clinically inapparent disease subtypes, in line with adult chronic lung disease imaging-based diagnosis (40,41). We thereby complement previous studies on neonatal MRI, which were based on proton density measurements (42), lung MRI relaxation times (19), and average signal intensities (12).

To highlight possibilities for diagnostic application, we demonstrated significantly increased performance in BPD classification when using our MRI lung features. Previous studies that solely relied on the performance of clinical variables achieved accurate BPD binary classification but showed only limited performance with regard to severity level stratification (4). The performance of our best binary model (AUC, 0.92 ± 0.02) exceeded previous radiation-free imaging-supported models (AUC, 0.83–0.86 [lung US] [9], 0.80 [lung MRI] [19]). Moreover, the inclusion of MRI lung features improved the identification of moderate and severe BPD cases (AUC, 0.84). Our multinomial BPD severity model (AUC, 0.75) outweighed the performance of 13 BPD classification models (AUC, 0.54–0.73) (4), resembling the performance of Ryan et al (43) (AUC, 0.76), despite this approach being dependent on oxygen supplementation as input for severity classification (4). Potential clinical value is especially supported by the good performance of the MRI lung features in a subset of extremely premature infants (GA, 25.4–28.6 weeks) in which clinical parameters do not sufficiently discriminate BPD severity. We additionally confirmed the value of automated MRI lung features for BPD grading, improving on previous studies estimating the duration of mechanical ventilation (15,18,43).

Our features outperformed standard radiomic features in the group of extremely premature infants (GA, 25.4–28.6 weeks), indicating that they contain complementary information for describing neonatal lung structure. As the combined application of both lung feature sets (radiomic and our lung features) further improved overall classification performance, novel approaches could benefit from considering comprehensive sets of automated MRI features to inform disease characterization.

Future studies need to address larger and more diverse annotated datasets to investigate the generalizability of our method, including studies to identify the required level of consistency for the imaging protocols and the consideration of different pathology patterns for translation into other forms of lung disease. Moreover, larger datasets could possibly enable the use of more complex DL architectures, such as the 3D U-Net, for which we found lower performance on our dataset. As BPD detection in this study was solely based on T2-weighted acquisitions, further improvement could be achieved by the integration of other imaging protocols including contrasts through proton density or T1 weighting. The use of two-dimensional multisection acquisitions with anisotropic voxel size in different respiratory states and planes can be advanced through ultrashort-echo-time pulse sequences (12,44).

In summary, our study contributes to the mounting evidence that artificial intelligence–driven MRI descriptors can serve as markers of lung disease in neonates, with the prospect of improving diagnostic processes by the use of a radiation-free imaging technique. We successfully demonstrated the effectiveness of artificial intelligence methods to generate quantifiable 3D MRI lung structural information, with the potential to improve precision for lung disease characterization in the challenging cohort of preterm neonates. Our results motivate future studies to further evaluate the clinical value of the models proposed, including their capability to guide therapeutic strategies and long-term monitoring.
